# Comparison of fimasartan and amlodipine therapy on carotid atherosclerotic plaque inflammation

**DOI:** 10.1002/clc.23133

**Published:** 2018-12-20

**Authors:** Minyoung Oh, Cheol Whan Lee, Jung‐Min Ahn, Duk‐Woo Park, Soo‐Jin Kang, Seung‐Whan Lee, Young‐Hak Kim, Dae Hyuk Moon, Seong‐Wook Park, Seung‐Jung Park

**Affiliations:** ^1^ Department of Nuclear Medicine, Asan Medical Center University of Ulsan College of Medicine Seoul South Korea; ^2^ Department of Medicine University of Ulsan College of Medicine Seoul South Korea

**Keywords:** amlodipine, fimasartan, plaque inflammation, positron emission tomography

## Abstract

**Background:**

The renin‐angiotensin system plays an important role in promoting atherosclerotic plaque inflammation, which may be inhibited by angiotension‐II receptor blockers.

**Hypothesis:**

We investigated the effects of fimasartan and amlodipine therapy on carotid atherosclerotic plaque inflammation using ^18^F‐fluorodeoxyglucose (^18^FDG) positron emission tomography (PET) imaging.

**Methods:**

Fifty patients with acute coronary syndrome (ACS) and at least one lesion with ^18^FDG uptake in the carotid artery (target‐to‐background ratio [TBR] ≥ 1.6) were randomly assigned to receive either fimasartan (60 mg once a day) or amlodipine (5 mg once a day). ^18^FDG PET examinations were performed in all patients at baseline and 6 months. The primary endpoint was the percent change in the index vessel TBR for the most diseased segment (MDS TBR).

**Results:**

The two groups had similar baseline characteristics. At the 6‐month follow‐up, index vessel and aorta MDS TBR significantly decreased in both groups. However, the percent change in index vessel MDS TBR was similar between the two groups (−9.33 ± 14.2% vs −7.73 ± 19.1%, respectively, *P* = 0.9). No significant difference was found for the percent change in the whole vessel TBR for the index vessel between the two groups, with similar findings for changes in MDS TBR or whole vessel TBR for the aorta. Total cholesterol, low‐density lipoprotein cholesterol levels, and blood pressure improved to a similar degree in both groups.

**Conclusions:**

Fimasartan and amlodipine reduce carotid atherosclerotic plaque inflammation similarly in patients with ACS, offering the same level of effectiveness.

## 1 INTRODUCTION

The renin‐angiotensin system (RAS) may promote atherosclerotic processes by the inducing of inflammation, endothelial dysfunction, and low‐density lipoprotein (LDL) oxidation.^1^, ^2^, ^3^ Several studies have shown upregulation of tissue angiotensin converting enzyme and angiotension‐II receptor type 1 (AT1 receptor) in human atherosclerotic plaques,^4^, ^5^ suggesting a potential role of tissue RAS in atherogenesis through local angiotension‐II effects.^6^, ^7^, ^8^ Angiotensin‐II may promote atherosclerotic plaque inflammation,^3^ and AT1 receptor blockers suppress plaque progression and induce plaque stabilization.^9^, ^10^ However, beneficial antiatherosclerotic properties of AT1 receptor blockers beyond blood pressure control have not yet been established. Although AT1 receptor blockers and calcium channel blockers are commonly used in patients with atherosclerotic cardiovascular disease, little is known about the comparative effects of these two agents on atherosclerotic plaque inflammation. Fimasartan, a potent AT1 receptor blocker, has been shown to have antiatherosclerotic effects in a rabbit model of atherosclerosis.^11^ We hypothesized that fimasartan is superior to amlopidine in reducing atherosclerotic plaque inflammation despite similar blood pressure reduction efficacy.

The present study compared the effects of fimasartan and amlodipine therapy on carotid atherosclerotic plaque inflammation in patients with acute coronary syndrome (ACS) using ^18^F‐fluorodeoxyglucose (^18^FDG) positron emission tomography (PET) imaging (FACE (Comparison of Fimasartan and Amlodipine Therapy on Carotid Atherosclerotic Plaque Inflammation) trial; ClinicalTrials.gov number, NCT02378064).

## 2 METHODS

### 2.1 Study design

This was a prospective, open label, randomized, single center trial conducted between May 2015 and December 2017. Patients were eligible if they presented with ACS, history of hypertension (or blood pressure ≥ 140/90 mm Hg at admission), carotid artery disease (diameter stenosis 20%‐50%), and at least one^18^FDG uptake lesion in the carotid artery (target‐to‐background ratio [TBR] ≥ 1.6) by^18^FDG PET/computed tomography (CT) imaging. Exclusion criteria included patients (a) scheduled for carotid endarterectomy or stenting, (b) with chronic disease that needed to be treated with oral, intravenous, or intraarticular steroid, (c) who had used RAS or calcium channel blocker therapy in the past 4 weeks, (d) with congestive heart failure or left ventricular ejection fraction less than 40%, (e) with chronic renal failure (serum creatinine >2.0 mg/dL or creatinine <40 mL/min [by Cockcroft‐Gault method], (f) with chronic liver disease, and (g) with type I diabetes.

### 2.2 ^18^FDG PET/CT examination

Baseline^18^FDG PET/CT scans were performed within 2 days of coronary angiography or percutaneous coronary intervention (3‐5 days after admission). Eligible patients meeting all the inclusion and none of the exclusion criteria were randomized at 1:1 ratio to receive either fimasartan (60 mg once a day for 6 months) or amlodipine (5 mg once a day for 6 months). All patients were required to take standard medications including antiplatelet agents and cholesterol‐lowering drugs and were requested to have a follow‐up ^18^FDG PET/CT examination at 6 months. Biochemical laboratory tests were also required at admission and at 6‐month follow‐up. The study protocol was approved by our Institutional Review Committee, and written informed consent was obtained from all patients.

All patients underwent vascular ^18^FDG PET/CT examination following standard methods.^12^, ^13^ Patients received intravenous ^18^FDG injection of (5.2 MBq [0.14 mCi]/kg body weight) after at least 8 hours fasting. Patients with diabetes mellitus kept their regular schedules of glucose controlling medications. Serum glucose levels were maintained under 130 mg/dL in all patients.

Three‐dimensional PET/CT scan was initiated 2 hours after ^18^FDG injection. First, CT was performed to correct photon attenuation and scattering without administration of contrast medium using a continuous spiral 64‐slice technique with 140‐kV and 200‐mA operating voltage and current, 0.98 pitch (39.4 mm/rotation), 2.5 mm slice thickness, and 0.4 seconds/rev a rotation. PET was performed immediately afterward, using a Discovery 690 PET/CT scanner (GE, Waukesha, Wisconsin) with 15.7‐cm axial field of view and 256 × 256 image reconstruction matrix. Patients were lying in the supine position using a head fixation device that held the patient's neck rigidly with respect to the shoulders. Images were acquired for 10 minutes in each bed position during normal tidal breathing, with overlapping scans from the skull base to the upper chest. After CT‐based attenuation and scattering correction, images were reconstructed using the three‐dimensional ordered subsets expectation maximization reconstruction algorithm (four iterations, 18 subsets) with time of flight reconstruction. PET data were routinely recalculated to provide images of standardized uptake values (SUVs) based on lean body mass.

### 2.3 Image analysis

Image analysis was performed on a dedicated workstation. ^18^FDG PET was quantified after identification of the ascending aorta, aortic arch, and common carotid arteries. PET images were visually evaluated for the presence of focal ^18^FDG uptake by the ascending aorta and carotid arteries. Arterial ^18^FDG uptake was determined by outlining a simple circular region of interest (ROI) around the artery on every slice of the co‐registered axial PET/CT images. On each image slice, the maximal SUVs of ^18^FDG in the ROI (containing the arterial wall and the lumen) were calculated as maximal pixel activity. The SUV was the decay corrected tissue concentration of ^18^FDG (in kBq/g), adjusted for lean body mass, and injected ^18^FDG dose. Maximal SUVs for each region were derived by averaging SUVs of all artery slices within an arterial territory. The SUVs were normalized to blood ^18^FDG activity by dividing them by the average blood ROI (calculated from at least six venous ROI measurements) estimated from the superior vena cava, yielding an arterial TBR.

The most diseased segment (MDS) TBR was measured by centering on the slice of the artery that showed the highest ^18^FDG activity, and then averaging five contiguous segments (approximately, 1.5 cm extent), combined with immediate inferior and superior neighbors. Whole vessel TBR was measured as the average of maximal TBR activity for all axial slices. Whole vessel ^18^FDG uptake (TBR) was measured in the three target arteries (right and left carotid and aorta) and used to indicate ^18^FDG defined atherosclerotic inflammation activity. Cardiac catheterization may have variable impact on ^18^FDG uptake of the ascending aorta due to catheter induced aortic injury, and the carotid artery with the highest ^18^FDG uptake at baseline was chosen as the index vessel.^13^

### 2.4 Study endpoints

The primary endpoint was the percent change in MDS TBR of the index vessel defined as (MDS TBR at 6 months—MDS TBR at baseline)/(MDS TBR at baseline) × 100. Secondary endpoints were changes in systolic/diastolic blood pressure, lipid profiles (total cholesterol, triglyceride, high‐density lipoprotein [HDL] cholesterol, and LDL cholesterol), and high sensitive C‐reactive protein.

### 2.5 Statistical analysis

Sample size of approximately 22 patients per treatment group was estimated to yield 90% power (assuming 15% SD in both fimasartan and amlodipine groups) for detecting a difference of 15% with a significance level of 0.05, using a two‐sided test. With 10% anticipated dropout rate, the planned enrollment was 25 patients per treatment group (total 50 patients). Continuous variables were expressed as means ± SDs or medians with interquartile ranges, whereas categorical variables were expressed as frequencies. Continuous variables were compared using the paired *t* test or Wilcoxon rank sum test for changes in each group, and unpaired *t* test or Mann‐Whitney *U* test for differences between groups. Statistical significance was defined as two‐sided *P* < 0.05.

## 3 RESULTS

**Figure 1:**
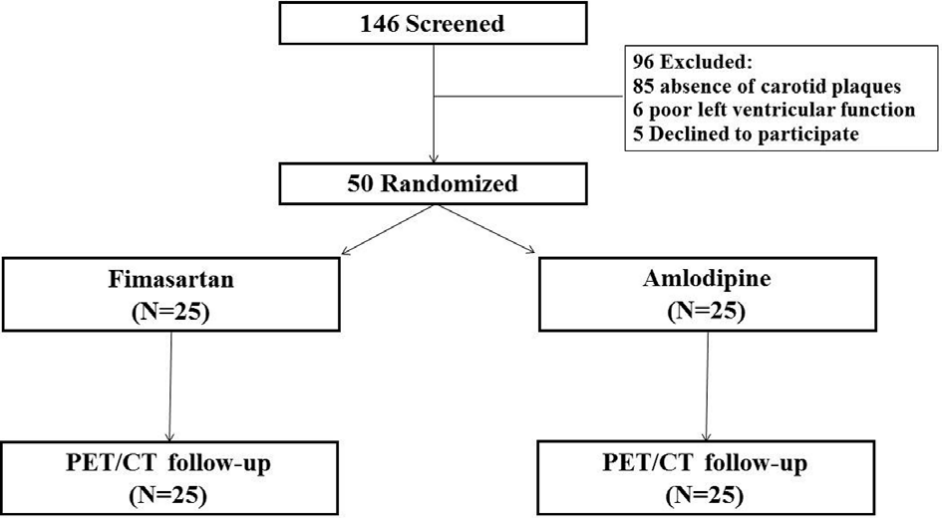
Patient enrollment. CT, computed tomography; PET, positron emission tomography

A total of 146 patients with ACS were screened for enrollment (Figure 1). Between May 2015 and June 2017, 50 patients who met all the eligibility criteria were randomly assigned to fimasartan or amlodipine groups. Exclusions were due to absence of carotid atherosclerosis (n = 85), poor left ventricular function (n = 6), and patient refusal (n = 5) (Figure 1). All patients completed PET/CT examination at 6‐month follow‐up. And there were no patients for each group who had chronic inflammatory conditions or had anti‐inflammatory medications associated with allergy, asthma, arthritis or inflammatory bowel disease.

Baseline characteristics were largely well balanced between the groups (Table 1). Median patient age was 60.0 years (interquartile range, 56‐67 years), and 86.0% were male. Clinical diagnosis at the time of admission was ST‐segment elevation myocardial infarction (76.0%), and non‐ST‐segment elevation ACS (24.0%). Most patients (98.0%) received percutaneous coronary intervention, except one patient (2.0%) with medication. For the patients with hypertension, there was no significant difference in duration between two groups (0.67 ± 1.30 vs 3.5 ± 5.15 years, *P* = 0.300). For the patients with smoking, there was no significant difference in pack years (38.8 ± 27.20 vs 30.0 ± 18.37 pack years, *P* = 0.730). Systolic and diastolic blood pressures at baseline and at 6‐month follow‐up were not significantly different between the two groups (Table 2). Changes of blood pressures were also similar (systolic: −20.0 ± 13.42% vs −12.2 ± 17.9%, *P* = 0.110; diastolic: −15.9 ± 16.69% vs −12.2.5 ± 17.86%, *P* = 0.460). Lipid profiles and high sensitivity C‐reactive protein levels at baseline were not significantly different between the two groups (Table 2). At 6‐month follow‐up, total cholesterol and LDL cholesterol levels significantly decreased in both groups (*P* < 0.001). High‐sensitivity C‐reactive protein levels significantly decreased in the fimasartan group (*P* = 0.017), but not in the amlodipine group (*P* = 0.068). Triglyceride and HDL cholesterol levels did not significantly change in both groups.

**Table 1:** Baseline clinical characteristics

Characteristic	Fimasartan (n = 25)	Amlodipine (n = 25)	*P*
Age (years)	61.8 ± 7.6	59.9 ± 8.8	0.781
Men	20 (80.0%)	23 (92.0%)	0.221
Current smoker	4 (16.0%)	5 (20.0%)	0.713
Diabetes mellitus	1 (4.0%)	4 (16.0%)	0.157
Hypertension^a^	12 (48.0%)	15 (60.0%)	0.395
Diagnosis			0.777
STEMI	18 (72.0%)	20 (80.0%)	
NSTE‐ACS	7 (28.0%)	5 (20.0%)	
Culprit artery of ACS			0.669
Left anterior descending coronary	18 (72.0%)	15 (60.0%)	
Left circumflex coronary	1 (4.0%)	1 (4.0%)	
Right coronary	6 (24.0%)	8 (32.0%)	
Ramus intermedius	0 (0%)	1 (4.0%)	
Culprit lesion PCI	25 (100.0%)	24 (97.5%)	0.312
Left ventricular ejection fraction (%)	54.6 ± 5.0	52.2 ± 9.6	0.291
Medications at the time of follow‐up			
Aspirin	25 (100%)	25 (100%)	1.0
P2Y12 inhibitors	25 (100.0%)	25 (100.0%)	1.0
β‐Blockers	19 (76.0%)	22 (88.0%)	0.269
Statins	25 (100.0%)	25 (100.0%)	1.0

Abbreviations: NSTE‐ACS, non‐ST‐segment elevation acute coronary syndrome; PCI, percutaneous coronary intervention; STEMI, ST‐segment elevation myocardial infarction.

History of hypertension.

**Table 2:** Laboratory findings

Characteristic	Fimasartan (n = 25)	Amlodipine (n = 25)	*P*
Systolic blood pressure (mm Hg)			
Baseline	148.8 ± 12.70	143.8 ± 12.30	0.158
6 months	125.5 ± 17.8	128.5 ± 15.5	0.536
Diastolic blood pressure (mm Hg)			
Baseline	81.0 ± 8.14	83.3 ± 11.56	0.416
6 months	70.9 ± 9.62	75.4 ± 11.90	0.152
Total cholesterol (mg/dl)			
Baseline	176.2 ± 33.73	176.2 ± 37.4	0.524
6 months	128.9 ± 18.79	136.1 ± 27.29	0.078
Triglyceride (mg/dl)			
Baseline	112.2 ± 46.04	113.1 ± 56.89	0.577
6 months	105.6 ± 32.02	123.32 ± 52.36	0.044
LDL cholesterol (mg/dl)			
Baseline	119.5 ± 32.4	118.3 ± 37.2	0.900
6 months	81.7 ± 15.5	86.7 ± 25.4	0.405
HDL cholesterol (mg/dl)			
Baseline	45.8 ± 9.2	45.4 ± 9.8	0.894
6 months	45.3 ± 9.9	46.6 ± 5.9	0.581
Hs‐CRP (mg/L)			
Baseline	0.33 ± 0.35	0.38 ± 0.72	0.289
6 months	0.10 ± 0.23	0.11 ± 0.19	0.877

Abbreviations: HDL, high‐density lipoprotein; Hs‐CRP, high sensitivity C‐reactive protein; LDL, low‐density lipoprotein.

**Figure 2:**
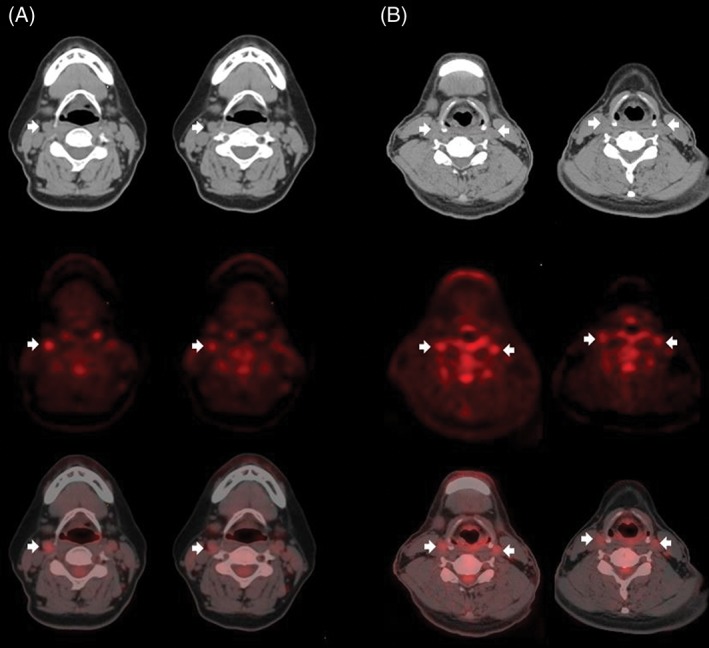
Index vessel ^18^FDG uptakes for a patient treated with (A) fimasartan or (B) amlodipine (arrows): (top) representative CT, (middle) ^18^FDG‐PET, (bottom) ^18^FDG‐PET/CT images at (left) baseline and (right) 6‐month follow‐up. ^18^FDG uptakes markedly decreased at the 6‐month follow‐up. CT, computed tomography; ^18^FDG, ^18^F‐fluorodeoxyglucose; PET, positron emission tomography

Figure 2 shows representative images of improved ^18^FDG uptake in carotid plaque after fimasartan or amlodipine therapy, and Table 3 shows ^18^FDG PET/CT data. Baseline measurements were not significantly different between the two groups. At 6‐month follow‐up, MDS TBR of the index vessel significantly decreased in both groups (*P* < 0.05). However, index vessel MDS TBR percent change (primary endpoint) was not significantly different (−9.33 ± 14.2% vs −7.73 ± 19.1%, respectively, *P* = 0.900). Whole vessel TBR of the index vessel significantly decreased in the fimasartan group (*P* = 0.019), but not in the amlodipine group (*P* = 0.152). Whole vessel TBR percent change of the index vessel was also not significantly different between the two groups. Similar findings were observed for changes in MDS TBR or whole vessel TBR of the aorta. There were no significant correlations between changes in lipid profiles or C‐reactive protein levels and percent changes in index vessel MDS TBR. Systolic and diastolic blood pressures were not correlated with percent change in index vessel MDS TBR.

**Table 3:** Changes in arterial inflammation activity

Characteristic	Fimasartan (n = 25)	Amlodipine (n = 25)	*P* between groups
MDS TBR of index carotid artery			
Baseline	2.36 ± 0.39	2.25 ± 0.48	0.379
Follow‐up	1.83 ± 0.37	1.81 ± 0.23	0.779
Nominal change	−0.21 ± 0.37	−0.23 ± 0.46	0.835
*P*‐value compared with baseline	0.009	0.019	
Percent change (primary endpoint)	−9.33 ± 14.2	−7.73 ± 19.1	0.900
Whole vessel TBR of index carotid artery			
Baseline	2.02 ± 0.33	1.92 ± 0.36	0.315
Follow‐up	1.83 ± 0.37	1.81 ± 0.23	0.213
Nominal change	−0.19 ± 0.37	−0.11 ± 0.38	0.482
*P*‐value compared with baseline	0.019	0.152	
Percent change	−8.0 ± 16.7	−2.81 ± 19.1	0.315
MDS TBR of aorta			
Baseline	2.58 ± 0.48	2.56 ± 0.42	0.892
Follow‐up	2.31 ± 0.46	2.32 ± 0.35	0.915
Nominal change	−0.28 ± 0.48	−0.25 ± 0.48	0.829
*P*‐value compared with baseline	0.009	0.017	
Percent change	−9.40 ± 16.8	−7.75 ± 18.0	0.739
Whole vessel TBR of aorta			
Baseline	2.49 ± 0.48	2.45 ± 0.40	0.784
Follow‐up	2.22 ± 0.47	2.23 ± 0.31	0.896
Nominal change	−0.27 ± 0.48	−0.22 ± 0.48	0.717
*P*‐value compared with baseline	0.010	0.030	
Percent change	−9.5 ± 16.8	−6.9 ± 18.8	0.739

Nominal change was calculated as follow‐up—baseline, and percent change as (follow‐up—baseline)/baseline × 100.

Abbreviations: MDS, most diseased segment; TBR, tissue blood ratio.

## 4 DISCUSSISON

Among patients with ACS and carotid artery disease, we found that MDS TBR of the carotid arteries and aorta significantly decreased in both fimasartan‐ and amlodipine‐treated patients. Whole vessel TBR of the index vessel significantly decreased in the fimasartan group, but not in the amlodipine group whereas those of aorta decreased in both groups. However, this effect did not differ significantly between the two groups, suggesting similar effects from both agents on atherosclerotic plaque inflammation. Improvement in total cholesterol, LDL cholesterol, and high sensitivity C‐reactive protein was also observed without between group differences.

Inflammation derives the atherosclerotic process, providing an important target for in‐vivo atherosclerosis imaging studies. ^18^FDG accumulates in atherosclerotic plaques in proportion to macrophage concentration, and arterial uptake correlates with arterial inflammatory burden.^12^, ^13^ The ^18^FDG PET signal is reproducible, providing a useful tool to assess serial changes of atherosclerotic plaque inflammation. Whole vessel TBR assesses both diseased and healthy segments, whereas MDS TBR reflects inflammatory activity of the diseased segment. The latter is more commonly used to evaluate therapeutic intervention impacts on atherosclerotic plaque inflammation. The present study showed that inflammatory indexes by ^18^FDG PET decreased equally in both groups, suggesting the drugs have similar effects on atherosclerotic plaque inflammation.

Antiatherosclerotic effects of RAS blockers appear to be independent of blood pressure reduction and may be in part due to RAS attenuation.^14^ Tissue RAS produces local angiotensin‐II that exerts various actions on the cardiovascular system.^15^ The ongoing telmisartan alone and in combination with ramipril global endpoint (ONTARGET) trial showed that telmisartan was an equally effective alternative to ramipril to prevent cardiovascular events.^16^ The impact of olmesartan on the progression of coronary atherosclerosis: evaluation by intravascular ultrasound (OLIVUS) trial showed that olmesartan led to a significant coronary plaque regression compared with the control group over the 14‐month follow‐up period.^10^ High‐dose fimasartan treatment suppressed atherosclerotic plaque development, lipid deposition, macrophage infiltration in a rabbit model of atherosclerosis.^11^ However, the present study showed no significant difference between fimasartan and amlodipine in reducing carotid atherosclerotic plaque inflammation assessed by ^18^FDG PET imaging. Such conflicting results may result from differences in drug doses between animal and human studies. In addition, the potential advantage of RAS blockers beyond antihypertensive efficacy for plaque modification in the setting of aggressive lipid lowering therapy still remains unclear. Statins were only used by 50% of OLIVUS trial patients, and LDL cholesterol levels were 104 mg/dL at follow‐up, which is above the current recommended limits. In contrast, all patients used statins in the current study, and LDL cholesterol levels were 82 mg/dL at 6‐month follow‐up. Intensive lipid lowering therapy may dilute RAS blocker effects on atherosclerotic plaque inflammation, leading to similar results in both groups.

Amlodipine is a long‐acting calcium channel blocker commonly used as an antihypertensive and antianginal, and anti‐inflammatory effects and antioxidant properties have been suggested.^17^ The prospective randomized evaluation of the vascular effects of norvasc trial (PREVENT) trial showed that amlodipine reduced carotid intima‐media thickness progression rate despite having no effect on angiographic progression of coronary atherosclerosis.^18^ The comparison of amlodipine vs enalapril to limit occurrences of thrombosis (CAMELOT) study showed that major adverse cardiovascular events were similarly reduced for both amlodipine and enalapril treated patients.^19^ The present study showed that atherosclerotic plaque inflammation similarly decreased in both groups with no blood pressure differences. These findings are inconsistent with those from other calcium channel blocker clinical trials,^20^ suggesting that amlodipine may have additional beneficial effects not mediated through blood pressure reduction.

Several classes of antihypertensive drugs have been used to control blood pressure, but optimal pharmacological agent choice is not yet fully established. Two clinical trials showed angiotensin converting enzyme inhibitor benefits for patients with coronary artery disease and normal or borderline blood pressure, whereas other trials showed no additional benefits beyond blood pressure.^6^, ^7^, ^8^ The antihypertensive and lipid lowering to prevent heart attack trial (ALLHAT) showed no differences of major cardiovascular events among lisinopril, diuretic, and amlodipine therapies.^21^ The valsartan antihypertensive long‐term use evaluation (VALUE) study showed similar event reduction for valsartan compared with amlodipine.^22^ The present study showed similar reductions of carotid or aorta inflammation, consistent with previous clinical trials that did not show superior outcomes for antihypertensive agents that modulate the RAS.

This study has several limitations. First, the analysis was limited by the small number of patients, which may have impacted the power to detect subtle differences in arterial inflammation. Second, our study was an open label, single center study, which is subject to inherent limitations. However, we tried to minimize these limitations by using blind ^18^FDG PET/CT evaluations.

## CONFLICTS OF INTEREST

The authors declare no potential conflict of interests.
